# AoCStream: All-on-Chip CNN Accelerator with Stream-Based Line-Buffer Architecture and Accelerator-Aware Pruning

**DOI:** 10.3390/s23198104

**Published:** 2023-09-27

**Authors:** Hyeong-Ju Kang, Byung-Do Yang

**Affiliations:** 1 School of Computer Science and Engineering, Korea University of Technology and Education, 1600 Chungjeol-ro, Dongnam-gu, Cheonan 31253, Republic of Korea; hjkang@koreatech.ac.kr; 2 School of Electronics Engineering, Chungbuk National University, 1 Chungdae-ro, Seowon-gu, Cheongju 28644, Republic of Korea

**Keywords:** convolutional neural networks, CNN accelerator, pruning

## Abstract

Convolutional neural networks (CNNs) play a crucial role in many EdgeAI and TinyML applications, but their implementation usually requires external memory, which degrades the feasibility of such resource-hungry environments. To solve this problem, this paper proposes memory-reduction methods at the algorithm and architecture level, implementing a reasonable-performance CNN with the on-chip memory of a practical device. At the algorithm level, accelerator-aware pruning is adopted to reduce the weight memory amount. For activation memory reduction, a stream-based line-buffer architecture is proposed. In the proposed architecture, each layer is implemented by a dedicated block, and the layer blocks operate in a pipelined way. Each block has a line buffer to store a few rows of input data instead of a frame buffer to store the whole feature map, reducing intermediate data-storage size. The experimental results show that the object-detection CNNs of MobileNetV1/V2 and an SSDLite variant, widely used in TinyML applications, can be implemented even on a low-end FPGA without external memory.

## 1. Introduction

Many EdgeAI and TinyML applications are related to computer-vision tasks like object detection, where convolutional neural networks (CNNs) are showing great performance [1,2,3,4,5,6,7,8,9]. However, CNNs require an enormous amount of memory and computation, so special hardware is usually adopted to implement them. In various kinds of CNN hardware, a CNN accelerator in ASIC or FPGA shows high efficiency.

Many CNN accelerators have been proposed [10,11,12,13,14,15], and some have focused on object detection [16,17,18,19,20,21,22]. One of the main concerns in designing a CNN accelerator is how to reduce the use of external memory. The processing of a CNN requires a large amount of memory, so the data are usually stored in an external memory like DRAM. Accessing an external DRAM consumes much power [23] and occupies long latency, which is a critical obstacle in the adoption of an edge application. Furthermore, adding a part largely affects the form factor of a small board like a TinyML environment.

There are two trivial solutions to this problem, namely embedding a large amount of on-chip memory [24,25,26] or using a very simplified CNN model [26]. However, these solutions are not so practical because of cost and degraded performance. It is still a challenging problem to reduce the required memory amount so that a CNN model frequently used for edge applications [27,28] can fit in the on-chip memory of a small device in an EdgeAI and TinyML environment like a low-end or mid-range FPGA device.

In this work, we reached this goal, all-on-chip implementation, by adopting two approaches. The CNN processing stores two kinds of data in memory, namely the weights and the intermediate activation data. To reduce the amount of weight memory, we exploit the pruning scheme [23,29,30,31], especially accelerator-aware pruning [32]. Pruning schemes can reduce the weight amount, but the irregularity leads to inefficient implementation. The accelerator-aware pruning prunes weights considering the base accelerator, so it does not harm the accelerator performance.

To reduce the amount of intermediate data memory, this work proposes a stream-based line-buffer architecture. The main component of a CNN is a convolutional layer. The proposed architecture is specialized to process a convolutional layer, storing only a few rows of intermediate data for each layer. A convolution is a local operation, so the calculation of an output activation requires only a few neighboring input data. If the input data are streamed into the processing block, only a few rows are required to be stored. To take full advantage of the line-buffer structure, a proper dataflow will be proposed, too. With the combination of the two schemes reducing the weight and the intermediate data memory, named AoCStream, an object-detection CNN widely used in EdgeAI and TinyML applications can be implemented in a low-end FPGA without an external memory.

This work is organized as follows. Section 2 introduces the basics of CNN computations, and Section 3 analyzes the memory sizes of CNN accelerators. The accelerator-aware pruning is introduced in Section 4, and the proposed architecture is described in Section 5. After the experimental results are shown in Section 6, related works are summarized in Section 7. Section 8 makes the concluding remarks.

## 2. Convolutional Neural Networks

A CNN consists of many layers, which are stacked from input to output. The data usually flow from input to output. The main layer in a CNN is a convolutional layer. A convolutional layer assumes *N* input feature maps whose height and width are *H* and *W*. A convolutional layer performs a convolution operation with K×K kernels on the input feature maps as described in the following equation and produces *M* output feature maps.

(1) $$\begin{array}{c} fo\left(m,y,x\right)=\sum_{n=0}^{N-1}\sum_{i=0}^{K-1}\sum_{j=0}^{K-1}w\left(m,n,i,j\right)\times fi\left(n,S\times y+i,S\times x+j\right)+bias\left(m\right), \end{array}$$

where fi() and fo() are a piece of the input and output feature map data, an input and output activation, respectively, w() is the weights, and *S* is the stride.

To reduce the amount of weight and computation, a convolutional layer can be divided into a depthwise convolutional layer and a point-wise convolutional layer [2], where a point-wise convolution is a normal 1 × 1 convolution. In the depthwise convolution, the number of the input feature maps, *N*, is equal to that of the output feature maps, *M*, and an output feature map is calculated from the corresponding input feature map as follows:

(2) $$\begin{array}{c} fo\left(n,y,x\right)=\sum_{i=0}^{K-1}\sum_{j=0}^{K-1}w\left(n,i,j\right)\times fi\left(n,S\times y+i,S\times x+j\right)+bias\left(n\right). \end{array}$$

CNNs are usually used for computer-vision tasks including object detection. One of the most popular CNN types for object detection is the single-shot multi-box detector (SSD) [5]. The SSD exploits an image classification CNN like VGG, ResNet, and MobileNet as a base CNN. The feature maps shrink further with auxiliary layers, and the detection box information is generated through a few more layers. There are some SSD variants, and SSDLite [3] uses depthwise convolutional layers instead of normal convolutional layers in the auxiliary part.

## 3. Memory Size of CNN Accelerators

One of the most important factors in designing CNN accelerators is the amount of required memory. Processing a neural network usually requires a huge amount of memory, usually larger than the on-chip memory size of a practical device. A CNN accelerator, therefore, usually uses external memory like DRAMs.

A CNN accelerator stores two types of data in memory, weights and intermediate activations. The amount of the weight memory is determined at the algorithm level by the CNN structure. The amount of the activation memory is also determined at the algorithm level, but it can be determined at the architecture level, too.

Traditionally, the memory amount for weights is believed to be much larger than that for the activations. In traditional CNNs, however, most of the weights belong to the fully connected layers [23]. The recent CNNs use only one or none fully connected layers [1,3], and the object-detection CNNs do not use fully connected layers at all [5,6,7]. In convolutional layers, the memory requirement for weights is not much larger than that for activations, compared to those in fully connected layers.

Furthermore, the activation amount is proportional to the square of the input image size. If the height and width of the input image are doubled, so are those of feature maps, and the activation amount increases by four times. This is not a big problem when the target application is the image classification because the input image size is usually very small, around 224. However, the modern object-detection CNNs use large input images varying from 300 [5] to 1280 [8]. In the current trend of processing larger input images, the activation amount will become larger in the future.

This activation amount directly affects the activation memory size of the conventional CNN accelerators, which usually exploit the frame-based architecture. In the architecture, a neural network is processed layer by layer. A whole input feature map is stored in a memory, called a frame buffer, and a CNN accelerator reads activations from the frame buffer, processes them, and stores the output activations. After generating the whole output feature maps, the CNN accelerator starts to process the next layer. Therefore, the CNN accelerator requires memory for the whole input or output feature maps, and the amount is doubled if the double buffering scheme is applied. Some structures process a few layers at the same time [13,33], but they store the intermediate data between the layer blocks, too.

**Figure 1:**
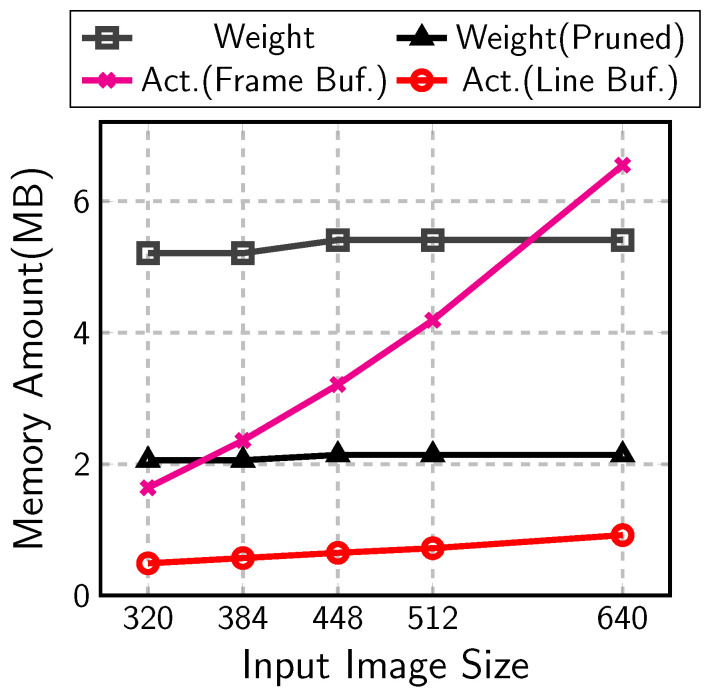
Memory amount for MobileNetV1 and SSDLiteX.

The memory sizes are analyzed in Figure 1 for the object-detection CNNs consisting of MobileNetV1 [2] and SSDLiteX [34], a variant of SSDLite. The CNNs are built for images with various sizes from 320 to 640. The number of auxiliary layer stages changes with the input sizes: four stages for the input sizes 320 and 384 and five stages for 448 to 640. The figure compares the memory amounts for each type of data with 8-bit quantization. For small input images, the memory amount for the activation frame buffer is around one third of that for the weights, which were denoted as *Act.(Frame Buf.)* and *Weight* in the figure, respectively. With large input images, however, the activations occupy almost the same memory as the weights do. Furthermore, the weight amount can be reduced by pruning.

## 4. Accelerator-Aware Pruning

Pruning is a scheme to reduce the number of weights and computation by forcing some weights to be zero [23,29,30,31,32]. Recent research on pruning shows the amount of weight can be reduced by three quarters in convolutional layers [23,32]. If the pruning is applied, the memory amount for weights is smaller than that for activations even with small input images (*Weight(Pruned)* in Figure 1).

There are two classes of pruning schemes: structured and unstructured. The structured pruning prunes weights in a regular pattern. A representative one is channel-wise pruning, where some whole kernels are selected and pruned. The unstructured pruning selects weights to be pruned with no regular patterns. Unstructured pruning can prune more weights than structured, but the irregular pruning pattern degrades the accelerator efficiency.

**Figure 2:**
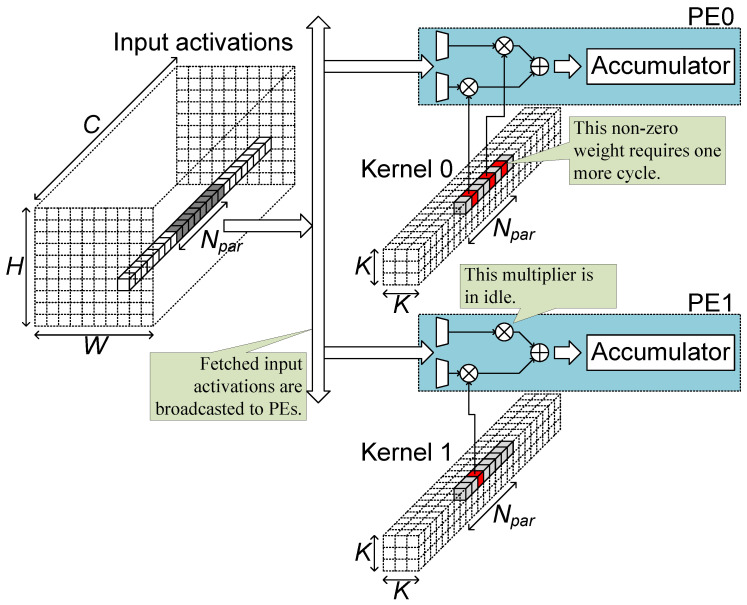
Convolutional layer processing with unstructured pruning.

Figure 2 illustrates a convolutional layer processing in a sparsity architecture like Cambricon-X [35] when the layer is pruned by unstructured pruning. In the accelerator structure of the figure, a group of $N_{par}$ input activations is fetched and broadcasted to processing elements (PEs). In the part of a kernel corresponding to the fetched activations, some weights are pruned to be zero (grey in the figure) and some weights are remained to be non-zero (red in the figure). Each PE has two multipliers and reads two non-zero weights from the weight memory. The PE selects activations corresponding to the non-zero weights and multiplies the weights and activations to sum and accumulate the results. In the unstructured pruning, there is no pattern, so in some kernels, three non-zero weights can be remained for the fetched group of activations, as shown in the upper kernel. Then one more cycle is required to process the third non-zero weight. In some kernels, only one non-zero weight can remain for the feature group of activations, as shown in the lower kernel, and then a multiplier in PE1 is in an idle state, doing nothing. Furthermore, for the additional cycle for the process of the third non-zero weight in the upper kernel, both multipliers in PE1 are in an idle state, too. The unstructured pruning leads to additional processing cycles and low multiplier utilization.

**Figure 3:**
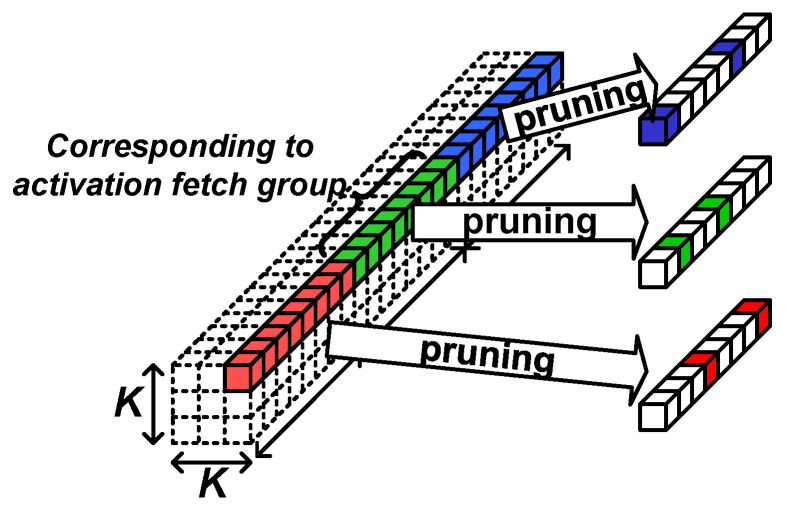
Accelerator-aware pruning.

**Figure 4:**
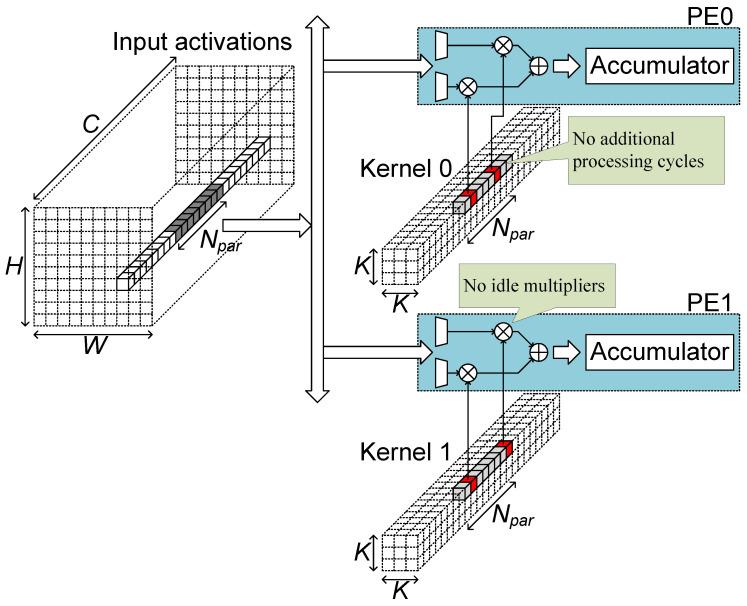
Convolutional layer processing with accelerator-aware pruning.

The algorithm part of the proposed AoCStream exploits the accelerator-aware pruning, which prunes weights with a regular pattern proper to an accelerator architecture [32]. As shown in Figure 3, the weights are pruned so that an equal number of weights remains for every activation group fetched together. With the accelerator-aware pruning, no additional processing cycles are required, and multipliers can be fully utilized, as shown in Figure 4. Even with the regular pattern, the pruning scheme can prune as much as the unstructured pruning without degrading the accelerator efficiency [32].

## 5. Stream-Based Line-Buffer Architecture

As mentioned in the previous section, the size of the weight memory can be reduced at the algorithm level with a method like pruning. However, there is no method to reduce the activation memory. The only way is by using a smaller input image despite the performance degradation or using another architecture. This section will focus on the reduction of the activation memory in the architecture and dataflow level, proposing a stream-based line-buffer architecture for all-on-chip CNN implementation, the architecture part of AoCStream.

### 5.1. Top Architecture

**Figure 5:**
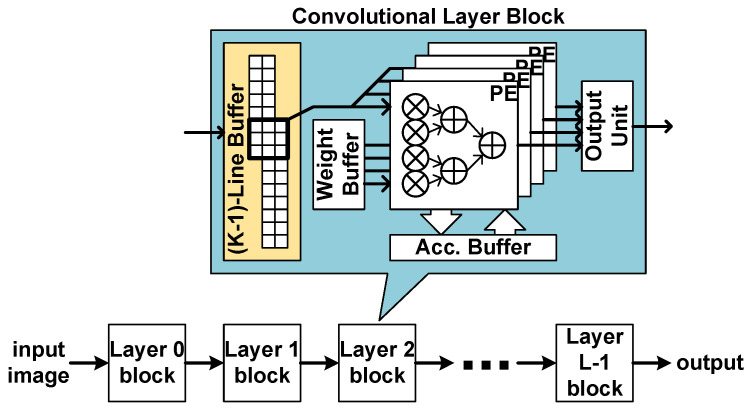
Stream-based line-buffer architecture.

The proposed accelerator processes a CNN in a layer-level pipelined way. Each layer has a corresponding processing block as shown in Figure 5. When a group of data are input to a block, the block processes the input data and generates a group of output data if possible. The generated group of data streams into the next block. Since each block does not wait for the previous block to complete the whole corresponding layer operation, all the blocks can operate in parallel. The structure of a layer block is determined by the corresponding layer type.

### 5.2. Convolutional Layer Block

The base operation of a convolutional layer is the two-dimensional convolution. In conventional image processing circuits, the two-dimensional convolution is usually processed by a stream-based structure with a line buffer of size (K−1) lines. In the structure, the input data are assumed not to reside in memory, but to stream in one by one. When one piece of input data streams in, the circuit processes the possible convolution operation.

As the typical image processing circuits, the proposed convolutional layer block uses the line buffer to hold the activations to be used with the input coming later. The current input data and the data in the line buffer are combined and broadcast to the processing elements (PEs), as shown in Figure 5. In the PEs, the activation data are multiplied with weights and accumulated with an accumulation buffer. When the accumulation is completed, the resulting data are quantized and output through the output unit.

The block has three memories, the line buffer, the weight memory, and the accumulation buffer. Theoretically, the shortest size of a line buffer is (K−1) lines. To reach this size, however, proper operation scheduling, called a dataflow, should be adopted.

### 5.3. Input-Centric Dataflow

There have been proposed many dataflows for the convolutional layer operation [11], but most of them assume the frame-based architecture. The main focus of the dataflows was to reduce the number of DRAM accesses by reusing the already-fetched data as many times as possible. However, such dataflows may increase the memory size in the all-on-chip implementation of this work.

To raise the possibility of the all-on-chip implementation, the memory size reduction should be focused on. As previously mentioned, the minimum line-buffer size is (K−1) lines. This line-buffer size, however, cannot be achieved by the previous reuse-focusing dataflows. To reach the size, the old input activations should be consumed with the current input activations as fast as possible.

**Figure 6:**
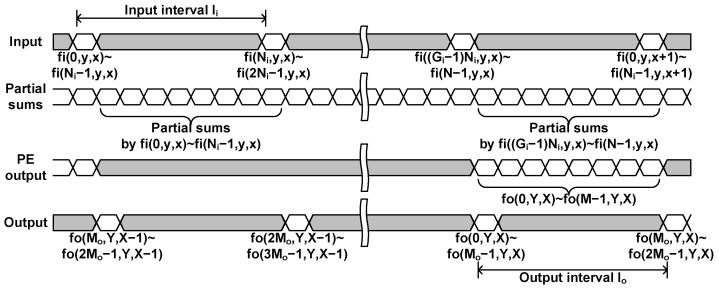
Convolutional layer processing timing with the input-centric dataflow.

For this purpose, this paper proposes the input-centric dataflow, where the operations that can be processed with the oldest input data in the line buffer are processed first. The input-centric dataflow assumes that the input data are streamed in the row-major order. For each spatial location, *N* channel data are divided into $G_{i}$ groups, and a group of $N/G_{i}=N_{i}$ data are streamed in together. Two consecutive groups are separated by the interval of $I_{i}$ cycles as shown in Figure 6.

**Figure 7:**
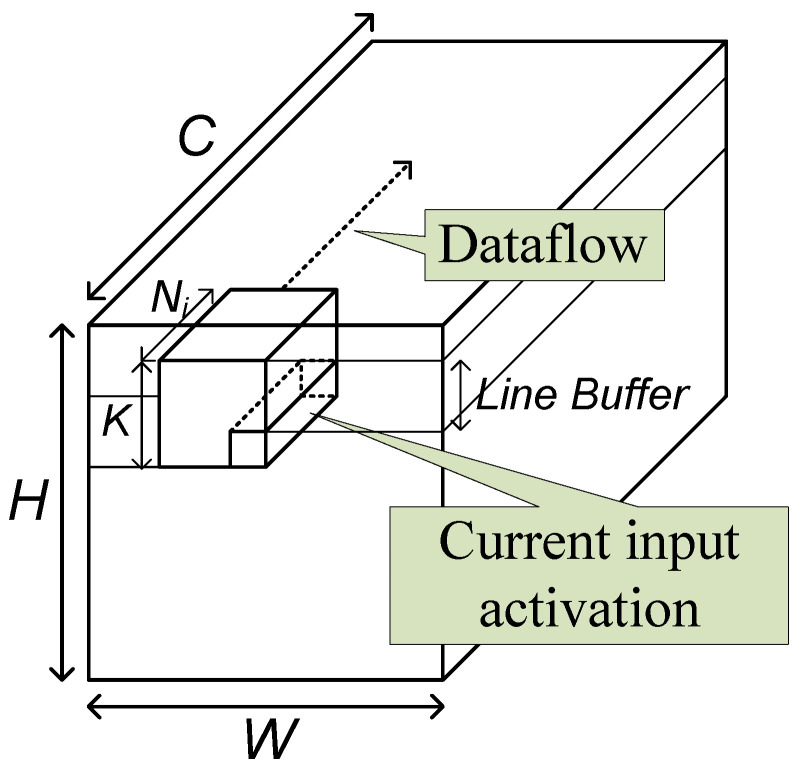
Input-centric dataflow.

With the $N_{i}$ data in a group, the layer block performs all the computations that can be done with the input data and the data stored in the buffer as shown in Figure 7. When *g*th group data, $fi\left(gN_{i},y,x\right)∼fi\left(\left(g+1\right)N_{i}-1,y,x\right)$, are input, the layer block calculates the following partial sums for outputs fo(m,Y,X), where 0≤m<M, Y=y−K+1, and X=x−K+1.

(3) $$\begin{array}{cc} fo_{g}\left(m,Y,X\right)=\sum_{n=gN_{i}}^{\left(g+1\right)N_{i}-1}\sum_{i=0}^{K-1} & \sum_{j=0}^{K-1}w\left(m,n,i,j\right)\times fi\left(n,Y+i,X+j\right), \end{array}$$

where the stride *S* is assumed to be 1 for simplicity, but the structure is not limited to that.

The partial sum requires $K\times K\times N_{i}\times M\times \left(1-r\right)$ MAC operations, where *r* is the pruning ratio, and the operations should be done in $I_{i}$ cycles. Therefore, the required number of MAC operators is $K\times K\times N_{i}\times M_{i}\times \left(1-r\right)$, where $M_{i}=M/I_{i}$. The layer block has $M_{i}$ PEs, and a PE with $K\times K\times N_{i}\times \left(1-r\right)$ MAC operators calculates a partial sum of the output at each cycle.

When a partial sum is calculated, it is accumulated with an accumulation buffer of size *M*. When all the data of a spatial location, fi(n,y,x) for 0≤n<N, are input through $G_{i}$ groups, the calculation of the output data, fo(m,Y,X) for 0≤m<M, is completed through the accumulation. The output data are collected at the output unit and streamed out in $G_{o}$ groups of $M/G_{o}=M_{o}$ data at the interval of $I_{o}$ cycles. If the spatial size of the input feature maps is equal to that of the output feature maps, the following relationship should be satisfied.

(4) $$\frac{N}{N_{i}}\times I_{i}\geq \frac{M}{M_{o}}\times I_{o}$$

### 5.4. Depthwise Convolutional Layer Block

The depthwise convolutional layer block also requires a line buffer of (K−1)-line size as the convolutional layer block in the previous subsection. In the depthwise convolution, the accumulation is not required between the input data groups. When $fi\left(gN_{i},y,x\right)∼fi\left(\left(g+1\right)N_{i}-1,y,x\right)$ data are input, we can calculate $fo\left(gN_{i},Y,X\right)∼fo\left(\left(g+1\right)N_{i}-1,Y,X\right)$. The required number of MAC operations is $K\times K\times N_{i}$. Each PE has a MAC unit, and the number of PEs should satisfy

(5) $$\mathrm{Number}\;\mathrm{of}\;\mathrm{PEs}\geq \frac{K\times K\times N_{i}}{I_{i}}.$$

In the proposed layer block structure, an output is designated to a PE. Therefore, the number of PEs should be a divisor of the number of outputs to be calculated. The number of PEs is determined under this constraint and Equation (5).

### 5.5. Pooling Layer Block

**Figure 8:**
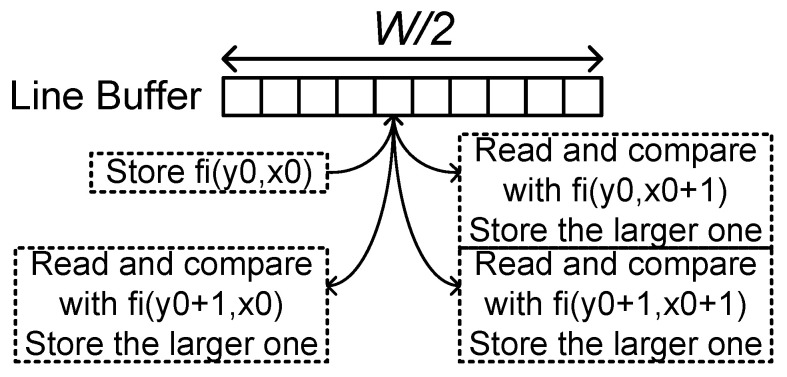
Pooling layer operation when *K* = 2 and *S* = 2.

The pooling layer block can be implemented in a similar way to the structure of the depthwise convolutional layer block. The maximum or average operators are used in the processing elements instead of multipliers and accumulators. The structure requires the line buffer of (K−1) lines and an output buffer for the data rate adjustment. However, for some configurations, the line-buffer size can be reduced. Figure 8 shows the max pooling layer operation when *K* = 2 and *S* = 2. When the block receives $fi(y_{0},x_{0})$ activations for even $y_{0}$ and $x_{0}$, the block stores the activations. With the activations on the next position, $fi(y_{0},x_{0}+1)$, the block reads the stored activations and compares them with the input data. The larger values are stored at the same line-buffer location. On the next row, the read-compare-write operation is repeated for $fi(y_{0}+1,x_{0})$ and $fi(y_{0}+1,x_{0}+1)$ on the same line-buffer location. Then the maximum value is stored in the line buffer. This operation flow only requires a line buffer of a half line.

### 5.6. Early-Delay Structure

Some of the modern CNNs use the inverted residual bottleneck scheme [3]. In the scheme, a bottleneck residual block consists of a 1 × 1 expansion layer, a *K* × *K* depthwise convolutional layer, and a 1 × 1 projection layer. If the input channel number is equal to the output channel number, a skip path is built between the input and the output. The expansion layer increases the number of channels by *t*-times, for example, six times in MobileNetV2.

**Figure 9:**
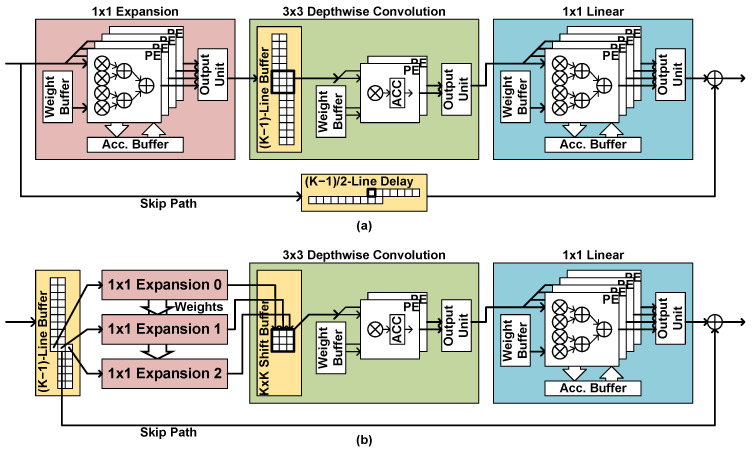
Bottleneck residual block implementation with (**a**) the naive structure and (**b**) the proposed early-delay structure.

To implement a bottleneck residual block, two line buffers are required, in the depthwise convolutional layer and the skip path, as shown in Figure 9a. If the input size of the residual block is N×H×W, the sizes of the line buffers are tN×W×(K−1) and N×W×(K−1)/2, respectively. The line buffers in the depthwise convolutional layer may occupy a major part of the activation memory because their sizes are *t*-times larger than the other line buffers.

To reduce the line-buffer size, this work proposes an early-delay structure, where the line buffer is placed in front of the expansion layer, as shown in Figure 9b. The number of channels is not tN but *N* at the input of the expansion layer, so the size of the line buffer is N×W×(K−1) instead of tN×W×(K−1), reducing the memory size greatly. However, the depthwise convolution requires the old input data as well as the current input data, so all the required data should be processed by the expansion layer together. In the proposed structure, the early-located line buffer outputs the current input data group with the (K−1) input data groups placed above the current input position. The *K* data groups are processed by the *K* expansion layer block, which have separate PEs with shared weight memory. The depthwise convolutional layer block receives *K* input data from *K* expansion layer blocks at the same time. The depthwise convolutional layer block has a *K* *K*-long shift registers, which supplies the required data to the PEs.

With the early-delay structure, the line-buffer size can be reduced by *t* times, and the delay line in the skip path can be removed, as shown in Figure 9b. Such memory size reduction comes with the increased number of operators in the expansion layer by *K* times. Table 1 compares the resource usage before and after the early-delay structure is applied to MobileNetV2 and SSDLiteX implementation for the 320 × 320 input image. Although the number of multipliers increases, the line-buffer size decreases much, providing the trade-off between the two resources, the internal memory and the operators. Since internal memory is usually a less-sufficient resource in CNN accelerators, especially in all-on-chip implementation, the early-delay structure can be a useful design choice. The inverted residual blocks are widely used in modern CNNs including MobileNetV3, EfficientDet, and MobileDet, so the proposed structure can be applied to those CNNs, too.

### 5.7. Architecture Comparison

#### 5.7.1. Memory Size

When a CNN is processed layer by layer as in the conventional frame-based architecture, a frame buffer is required to store the input and output feature maps. For a layer *l*, a frame buffer of size $H_{l}\times W_{l}\times N_{l}$ is required for the input feature maps, and another of size $H_{l+1}\times W_{l+1}\times N_{l+1}$ is required for the output feature maps. Since a frame buffer can be reused between layers, the maximum size is required as follows:

(6) $$\mathrm{Frame}\;\mathrm{Buffer}\;\mathrm{Size}=\max_{l}H_{l}\times W_{l}\times N_{l}$$

In the proposed architecture, a line buffer is used in each convolutional layer block, depthwise convolutional layer block, and pooling layer block. Since the blocks operate in parallel, the line buffers cannot be shared. Therefore, the total size of the line buffers is

(7) $$\mathrm{Line}\text{-}\mathrm{Buffer}\;\mathrm{Size}=\sum_{l}\left(K_{l}-1\right)\times W_{l}\times N_{l}.$$

When the input image size is scaled up, the input image is enlarged vertically and horizontally. The frame-buffer size in Equation (6) increases with the square of the scale. On the contrary, the line-buffer size in Equation (7) has only the width term, $W_{l}$. The line-buffer size is proportional to the scale linearly. In Figure 1, the frame-buffer size, *Act.(Frame Buf.)*, increases rapidly with the input image size. However, the line-buffer size, denoted as *Act.(Line Buf.)*, increases much slowly to be less than one quarter of the frame-buffer size at the 512 × 512 input image case.

**Figure 10:**
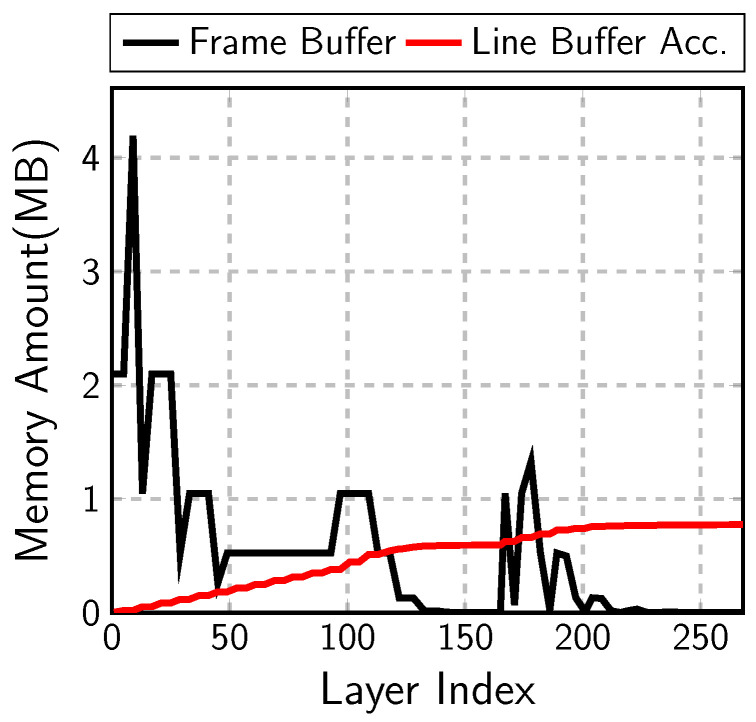
Frame-buffer vs. line-buffer size.

Figure 10 compares the frame-buffer size and the accumulated line-buffer size in each layer of MobileNetV1 and SSDLiteX with 512 × 512 input image. The maximum size of the frame buffer is 4 MB at the output of the first point-wise convolution. The line buffer in each layer is very small, so it would not be clearly shown in the figure. Instead of the line-buffer size in each layer, the figure illustrates the accumulated line-buffer amount, which is less than one quarter of the frame-buffer size.

#### 5.7.2. Latency

**Figure 11:**
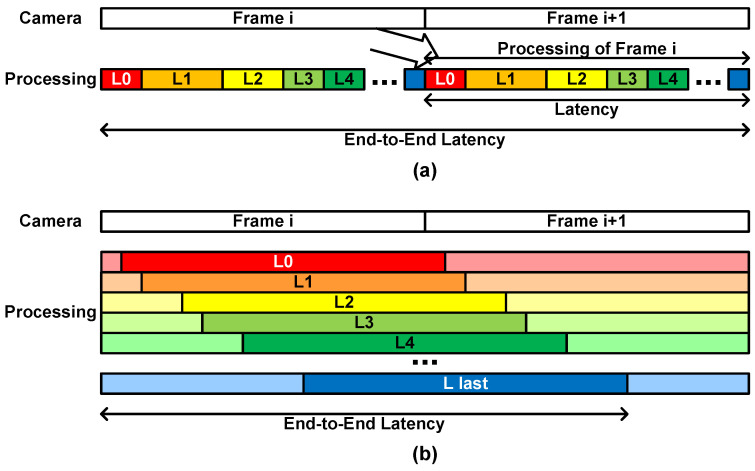
Latency of (**a**) the frame-based architecture and (**b**) the proposed architecture.

The frame-based architecture begins processing of a frame after the whole image is stored from a camera image stream. After processing all the layers one by one, the accelerator can process the next image, as shown in Figure 11a. If the latency is defined as the time between the start and the end of the processing, as commonly used in the frame-based architecture, the latency is closely related to the throughput as follows:

(8) $$\begin{array}{c} \mathrm{Throughput}=\frac{1}{\mathrm{Latency}} \end{array}$$

However, if we consider the latency from the beginning of the frame input to the completion of the processing, the end-to-end latency is the sum of the time of storing the input image frame and that of the CNN processing. It becomes two image frame periods.

In the layer-wise pipelined architecture as the proposed one, each layer block does not wait for the previous layer block to complete the processing of the assigned layer. It performs the layer processing as soon as it receives the necessary data. Each layer block occupies a certain amount of latency, summing into the whole latency, as shown in Figure 11b. Therefore, the end-to-end latency is determined by the CNN model structure and the architecture details. The experimental results show that the CNN processing completes at around the 280th line of the next image input when the MobileNetV1 and SSDLiteX are applied with the image size 512. The end-to-end latency is around one and a half image frame periods.

### 5.8. All-on-Chip Accelerator

The weight pruning and the line-buffer architecture reduces the storage of the weights and the intermediate data, so their combination, known as AoCStream, can lead to all-on-chip implementation. The affordable amount of on-chip resources may vary depending on the target environment. In this work, we will use the resource amount of low-end or mid-range FPGA devices as a criterion. If directly used for accelerator implementation, such a device may be a practical choice for an EdgeAI and TinyML environment. FPGAs are also widely used for a prototype before ASIC manufacturing, where the implementation on such small FPGA devices may indicate the feasibility of a small-sized chip.

As an example, the 512 × 512 input image case in Figure 1 requires a weight memory of around 5 MB and the frame buffer memory of around 4 MB. The total memory requirement of 9 MB cannot be afforded by a low-end or mid-range FPGA device like Xilinx XCKU5P, whose on-chip memory size is 4 MB. The two proposed schemes, the accelerator-aware pruning and the line-buffer architecture, can reduce the memory size by around three quarters. The total memory size becomes around 2.9 MB, which is less than the on-chip memory size of XCKU5P. Furthermore, the 320 × 320 input image case requires 2.5 MB, which is affordable in an even smaller device like XC7K325T.

The proposed scheme does not guarantee that any CNN can be implemented with the on-chip memory of any device. There will be no such method. The proposed method, however, broadens the possibility of the all-on-chip implementation, higher performance CNNs on smaller devices for resource-hungry environments.

## 6. Experimental Results

**Figure 12:**
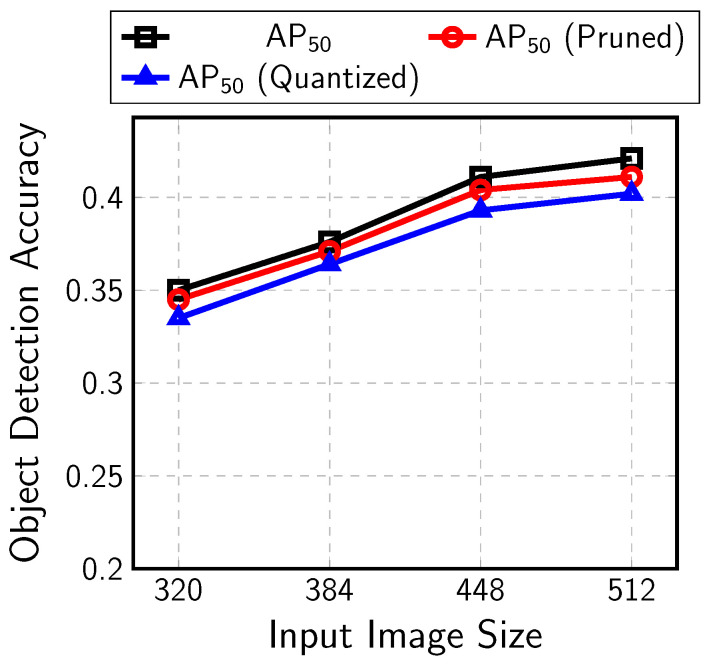
Object-detection accuracy for MS COCO dataset.

Object-detection CNNs based on MobileNetV1/V2 and SSDLiteX with various input sizes were trained and implemented with the proposed AoCStream architecture. The CNNs were trained with the MS COCO data set and pruned by the accelerator-aware pruning. The pruning ratio is 62.5% or 75%, which means five or six weights are pruned for every eight weights along the channel axis. The pruned CNNs were quantized with 8–10 bits without fine-tuning. The object-detection accuracy, $AP_{50}$, for the MS COCO dataset is provided after each step of training, pruning, and quantization in Figure 12. Pruning and quantization degrade $AP_{50}$ by around 0.01–0.02, but the detection accuracy is still high for such compact CNNs. If retraining is applied with quantization, better detection accuracy could be obtained. The final AP values are shown in the fifth row of Table 2.

The AoCStream accelerator was designed at the register-transfer level (RTL) for the quantized CNNs and implemented for a low-end Xilinx FPGA, XCKU5P, which is the second smallest device in the UltraScale+ Kintex series with 4 MB on-chip memory. The implementation results are shown on Table 2. The table describes the occupancy of FPGA resources including look-up tables (LUT), registers, block memory (BRAM), ultra memory (URAM), and operation units (DSP).

The last five rows of the table show the maximum operating clock frequency, the throughput in frames per second (FPS), two DSP efficiencies, and the external memory use. The first DSP efficiency is calculated by

(9) $$\begin{array}{c} \mathrm{DSP}\;\mathrm{Efficiency}\;1=\frac{\left(\mathrm{Operations}/\mathrm{Frame})\times (\mathrm{Frames}/\mathrm{second}\right)}{2\times (\mathrm{Number}\;\mathrm{of}\;\mathrm{DSPs})\times (\mathrm{Clock}\;\mathrm{Freq}.)}, \end{array}$$

where the 2× in the denominator reflects that a DSP can process two operations, a multiplication and an addition, simultaneously. The second DSP efficiency is the effective efficiency, which includes zero-skipped operations in a sparsity architecture, so the effective efficiency can be higher than 100% if pruning is applied. The last *Ext. Mem.* row with *None* indicates that the proposed architecture can store the whole intermediate data and weights on the on-chip BRAM and URAM even for the input image size 512 × 512. The table also shows that the all-on-chip implementation leads to high throughput and efficiency. The architecture can process images at 90 to 260 FPS, which is much faster than the real-time speed of 30 FPS. The implementation of MobileNetV2 and SSDLiteX shows a somewhat lower efficiency, but this is because of the duplicated operations for the early-delay structure.

Some of the previous architectures for a CNN accelerator are compared in Table 3 and Table 4. For a fair comparison, the proposed architecture is implemented on two older 7-series FPGAs, whose on-chip memory sizes are 2 MB and 4.6 MB. Because of the resource limitation, some layers are pruned to 87.5% for XC7K325T. At the last *Ext. Mem.* row, *W* and *A* means the weights and the activations are stored in external memory, respectively.

In Table 3, the AoCStream architecture is compared with the previous frame-based architectures. Those architectures use CNNs similar to the one used in this work. The second-column architecture used the MobileNetV1 and SSD combination [17], and its throughput is the highest in the previous ones but similar to that of the proposed. Furthermore, it uses around five times more DSPs. The architecture of the third column used MobileNetV2 and SSDLite with a small input size [16]. Despite such a small input size and high DSP usage, the throughput is very low. Since the two architectures do not exploit the pruning scheme, they require more multipliers than the proposed architecture. Furthermore, the two accelerators are based on the frame-based architecture, so their DSP efficiency is very low because of the DRAM accesses, leading to more DSP unit occupancy.

**Figure 13:**
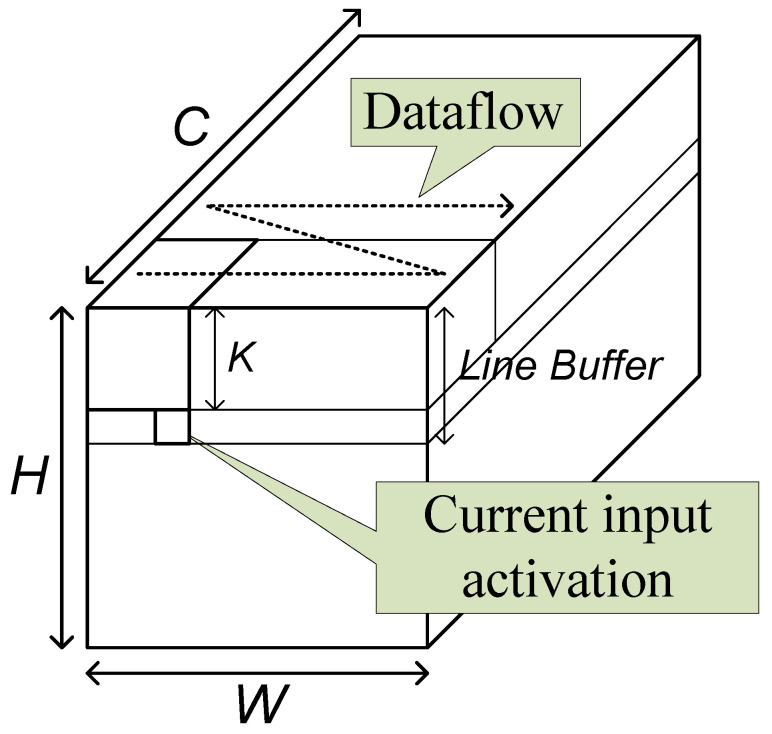
Dataflow of [18].

Table 4 compares the AoCSctream with a previous accelerator exploiting a line-buffer architecture similar to the proposed one. Since the accelerator used a dataflow focusing on the weight reuse as shown in Figure 13, the size of line buffers is (K+1) lines. In [18], YOLOv2-Tiny was implemented with one-bit weight quantization, and the results are presented in the second column of the table. The same CNN with the same configuration, no pruning, and one-bit weight quantization, is also implemented with the proposed AoCStream architecture as described in the fourth column. Because of one-bit quantization, no DSPs are used in the convolutional layers. The table shows the AoCStream architecture reduces the usage of the resources, especially that of BRAM by 40%. YOLOv2-Tiny with pruning and multi-bit quantization is also implemented in the fifth column, showing higher accuracy with more resources.

In the third column of the table, an accelerator using YOLOv3 of [19] is compared, and it still needs an external memory for weights because of larger line buffers. Because it used a different CNN, it is difficult to directly compare it with the proposed one, but its DSP efficiency is very low. Even though they adopted a sparsity architecture, the effective DSP efficiency is not higher than 100% probably because of DRAM accesses for weights.

HPIPE also used a line-buffer architecture [22], but it was implemented on a different type of FPGA. Because of the different internal FPGA structure, it is not compared in the table. However, HPIPE uses much more resources to implement a similar object-detection CNN, 4434 DSPs and 7179 M20K BRAMs for MobileNetV1 and SSD, showing less than 50% DSP efficiency. Such resource requirement is not suitable for edge applications.

## 7. Related Works

Many CNN accelerators have been proposed, but most of them have the frame-based architecture [10,11,12,13,14,15,16,17,20,21]. Such architectures require an external DRAM, and their operator efficiency is low because of the DRAM access delay. Because the frame-buffer size is proportional to the square of the input size, the architectures are not appropriate for object detection in an EdgeAI and TinyML environment.

Much smaller number of works have used a line-buffer architecture for CNNs [18,19,22,26,36,37,38]. However, they did not employ a dataflow proper to the line-buffer structure. Their dataflow focuses on the weight data reuse, processing *K* lines above the current input row as shown in Figure 13. Those dataflows lead to the larger line-buffer of size *K* or (K+1) lines. The large line buffers make their accelerators use external memory for the weights [18,19,36] or require a very large FPGA device [22,26].

The proposed dataflow reuses the input feature map data as much as possible. After a $K\times K\times N_{i}$ input activation data block is gathered, the PEs perform all the computations related to the block. This dataflow property enables the line-buffer size of (K−1) lines. However, this dataflow cannot reuse the weights, so it is proper to have a structure with all the weights in the on-chip memory.

## 8. Conclusions

In this paper, object-detection CNNs with reasonable performance were implemented only with the on-chip memory of a practical device suitable for EdgeAI and TinyML environments. The memory amount is reduced at the algorithm level, accelerator-aware pruning, and at the architecture level, a stream-based line-buffer architecture. In the architecture, a dedicated block is assigned to each layer, and the layer blocks operate in a pipelined way. The intermediate data are streamed into and out of each block, so only a few rows are stored in each block due to the property of the convolution operation. The reduction of the intermediate data storage is combined with the reduction of the weight storage by pruning to remove the need for external memory. The all-on-chip implementation greatly enhances the performance of the CNN accelerator. The architecture can be applied to various CNNs for other computer-vision tasks in edge applications.

## Figures and Tables

**Table 1:** Effect of early-delay structure.

Resource	Before	After
Line Buffer (KB)	487	224
Acc. Buffer (KB)	60	129
MAC Operators	402	582

**Table 2:** FPGA implementation results of the proposed architecture.

Architecture	AoCStream (Proposed)				
CNN	MNetV1 + SSDLiteX				MNetV2 + SSDLiteX
Input Size	320	384	448	512	320
Pruning Rate	75%	75%	75%	75%	62.5%
MS COCO AP	0.211	0.231	0.247	0.253	0.203
FPGA	XCKU5P				
LUT (K)	145	145	154	148	169
Reg (K)	219	219	233	232	298
BRAM	454	454	476	476	361
URAM	20	20	20	36	51
DSP	458	458	468	468	588
Clock (MHz)	428	420	393	403	373
Throughput (FPS)	261.3	178.2	122.5	96.0	227.7
DSP Efficiency 1 (%)	81.2	81.2	79.7	79.7	42.4
DSP Efficiency 2 (%)	292.6	292.5	286.9	286.9	99.6
Ext. Mem.	None	None	None	None	None

**Table 3:** FPGA implementation comparison with previous frame-based architectures.

Architecture	[17]	[16]	AoCStream (Proposed)
CNN	MNetV1	MNetV2	MNetV1
	+SSD	+SSDLite	+SSDLiteX
Input Size	320	224	320
MS COCO AP	0.193	0.203	0.206
FPGA	XCZU9EG	ZC706	XC7K325T
LUT (K)	162	148	151
Reg (K)	301	192	194
BRAM	771	311	433
DSP	2070	728	354
Clock (MHz)	333	100	174
Throughput (FPS)	124.3	15.4	94.5
DSP Efficiency 1 (%)	22.3	8.3	77.4
DSP Efficiency 2 (%)	-	-	337.8
Ext. Mem.	WA	WA	None

**Table 4:** FPGA implementation comparison with previous line-buffer architectures.

Architecture	[18,19]		AoCStream (Proposed)		
CNN	YOLOv2	YOLOv3	YOLOv2	YOLOv2	MNetV1
	Tiny		Tiny	Tiny	+SSDLiteX
Input Size	416	416	416	416	512
VOC mAP	0.514	-	0.514	0.529	-
MS COCO AP	-	0.310	-	-	0.253
FPGA	XC7VX485T				
LUT (K)	86	230	36	54	161
Reg (K)	60	223	47	62	235
BRAM	513	972.5	310.5	768.5	769
DSP	168	2640	9	272	468
Clock (MHz)	200	200	249	244	216
Throughput (FPS)	66.56	11.66	90.0	88.0	51.6
DSP Efficiency 1 (%)	-	-	-	89.5	79.7
DSP Efficiency 2 (%)	-	72.4	-	328.1	286.9
Ext. Mem.	-	W	None	None	None

## Data Availability

The implement results are available in https://github.com/HyeongjuKang/AoCStream (accessed on 14 August 2023).

## Abbreviations

The following abbreviations are used in this manuscript:

ASIC	Application Specific Integrated Circuit
FPGA	Field Programmable Gate Array
DRAM	Dynamic Random Access Memory
MAC	Multiplication and Accumulation
MS COCO	Microsoft Common Objects in Context
VOC	PASCAL Visual Object Classes
AP	Average Precision
